# Oligodendrocyte arrangement, identification and morphology in the developing superior olivary complex

**DOI:** 10.3389/fncel.2025.1561312

**Published:** 2025-03-28

**Authors:** Alina Carola Zacher, Melissa Grabinski, Laura Console-Meyer, Felix Felmy, Christina Pätz-Warncke

**Affiliations:** ^1^Institute for Zoology, University of Veterinary Medicine Foundation, Hannover, Germany; ^2^Hannover Graduate School for Neurosciences, Infection Medicine and Veterinary Sciences (HGNI), Hannover, Germany

**Keywords:** oligodendrocyte distribution, oligodendrocyte marker, oligodendrocyte morphology, superior olivary complex, postnatal development

## Abstract

Oligodendrocytes provide myelination, metabolic and developmental support for neurons and circuits. Within the auditory superior olivary complex (SOC), relevant for sound localization and spectro-temporal integration, oligodendrocytes are fundamental for fast neuronal communication and accurate timing of sound signals. Despite their important role in function and development, an assessment of their developmental arrangement and morphology is missing for the SOC. Here, immunofluorescence labeling and single cell electroporation was used to quantify their distribution, identification and morphology between postnatal day (P) 5 and ~ P54 in the SOC of Mongolian gerbils (*Meriones unguiculatus*). Oligodendrocytes show developmental, region-specific accumulations, redistributions and density profiles. Their identification by Olig2 and SOX10 appears age specific, while myelinating oligodendrocytes are detected by co-labeling with S100 irrespective of age. Comparison of oligodendrocyte density and identification between mature gerbil and Etruscan shrew (*Suncus etruscus*), revealed species-specific differences. Morphologically, the number of myelinating processes decreased, while process length, diameter and coverage area of oligodendrocytes increased during development. Together, oligodendrocyte developmental alterations occur at moments of SOC circuit refinement supporting functions beyond myelination.

## 1 Introduction

Oligodendrocytes are the myelinating cells of the central nervous system and are found in all vertebrates (Baumann and Pham-Dinh, 2001; Waehneldt, 1990). In most mammals, myelination occurs from late embryonic to early postnatal ages and depends on brain area and the molecular interactions between neurons, axons and oligodendrocytes (Baumann and Pham-Dinh, 2001; Duncan et al., 2021; Rogister et al., 1999; Tomassy et al., 2016). Functionally, the myelin sheath lowers the axons membrane capacitance and increases membrane resistance (Castelfranco and Hartline, 2015) promoting fast propagation of action potentials (Nave and Werner, 2014) and, thus, facilitates efficient and precise transfer of neuronal communication. In addition, oligodendrocytes provide metabolic support influencing the neurons viability, ion and water homeostasis, and thereby affect memory and learning (Fünfschilling et al., 2012; Kuhn et al., 2019; Lee et al., 2012; Monje, 2018; Philips and Rothstein, 2017; Stadelmann et al., 2019).

Neurons of the superior olivary complex (SOC) rely on fast and precise action potential propagation to facilitate temporal processing of auditory cues (Oertel, 1999) relevant for, e.g., binaural detection (Grothe et al., 2010). The SOC is a group of brainstem nuclei comprising the medial nucleus of the trapezoid body (MNTB), the medial superior olive (MSO) and the lateral superior olive (LSO). The MNTB projects well-timed inhibition to the MSO and LSO, which accomplish sound source localization by detecting interaural time and level differences (ITD and ILD; Goldberg and Brown, 1969; Boudreau and Tsuchitani, 1968). Sound source localization requires sub-millisecond integration (Klumpp and Eady, 1956; Leibold and Grothe, 2015) relying on precisely timed inputs. This detection process faces anatomical challenges because ipsilateral and contralateral axons must travel different distances but synaptic input to binaural detector neurons must be matched with exquisite timing (Long et al., 2018). A key mechanism for matching traveling times is the adaption of myelin thickness and length of myelin segments (Li et al., 2022; Seidl and Rubel, 2016). In the MNTB, accurate myelination is crucial for rapid and precise action potential propagation (Kim et al., 2013) and is influenced by sensory activity (Sinclair et al., 2017; Stancu et al., 2024) on an axon-to-axon-basis (Stancu et al., 2024). Moreover, oligodendroglial brain-derived neurotrophic factor (BDNF) signaling regulates presynaptic release properties at calyx terminals (Jang et al., 2019). Thus, oligodendrocytes play a crucial role within the circuitry relevant for temporal processing and binaural hearing (Cramer and Rubel, 2016).

Myelinating oligodendrocytes develop from neural stem cell-derived oligodendrocyte progenitor cells (OPCs), which migrate from the subventricular zone (Rogister et al., 1999; van Tilborg et al., 2018) into their final destination where they mature and differentiate (Baumann and Pham-Dinh, 2001; Michalski and Kothary, 2015; Simons and Nave, 2015). During the myelination process, excess oligodendrocytes are produced and undergo apoptosis if they fail to contact an appropriate axon (Barres et al., 1992; Barres and Raff, 1999; Trapp et al., 1997). Thus, the final number of myelinating oligodendrocytes matches the number and length of axons that need to be myelinated (Barres and Raff, 1999; McTigue and Tripathi, 2008). The timely differentiation of OPCs can occur independent of their axonal targets and might be region-specific (Almeida and Lyons, 2016). However, not all OPCs mature or undergo apoptosis. Some OPCs persists throughout development (Dawson et al., 2003; Hughes et al., 2013), as shown in the mouse MNTB (Lucas et al., 2021), resulting in oligodendrocytes of different maturation stages in the adult brain. Therefore, the oligodendrocyte population in the mature brain shows a large heterogeneity at least in their morphological appearance (Marques et al., 2016; Osanai et al., 2022). The proportion of putative OPCs and myelinating oligodendrocytes and possible region-specific differences in the developing SOC nuclei have not yet been described.

The timing of myelin establishment and the associated appearance of oligodendrocytes mirrors key stages of auditory circuit refinement (Long et al., 2018). In the MNTB of rodents, oligodendrocytes can be already found at P0 increasing in number during development (Brandebura et al., 2018; Dinh et al., 2014; Kolson et al., 2016). Myelin, however, can be detected only from P7/P8 on (Leao et al., 2005; Nabel et al., 2024; Saliu et al., 2014) and is highly expressed at MNTB axons around P13/P14 (Kim et al., 2013; Nabel et al., 2024), shortly after hearing onset (P11/12: Finck et al., 1972; Ryan et al., 1982; Smith and Kraus, 1987). Mature-like myelination is reached between P25 and P35, at least in gerbil (Sinclair et al., 2017). In the LSO, myelin basic protein expression starts during the first postnatal week, increases until shortly after hearing onset and appears consistent thereafter (Hafidi et al., 1996). Thus, in the SOC, the onset of myelination coincides with the synaptic and circuit refinement (Kandler et al., 2009). While data exist on oligodendrocyte occurrence in the MNTB, the influence of developmental changes in cell count and size of SOC nuclei (Simmons et al., 1999; Zacher et al., 2023) on cell density remains unclear. Also, a coherent quantitative analysis over the early and late postnatal period and nucleus-specific differences in the SOC are missing.

We therefore quantified oligodendrocyte distribution, number and density from P5 to P59 for the MNTB, MSO, LSO, a region dorsal to the LSO centered between the medial and lateral limb (dLSO) and a control area outside of the SOC in Mongolian gerbil. We observed that in the developing SOC oligodendrocytes accumulate and redistribute locally and temporarily. Oligodendrocyte density and identification varied between gerbil and Etruscan shrew indicating species-specific differences. We found that S100-labeling allows differentiation and quantification of non-myelinating and myelinating oligodendrocytes revealing that during development mostly non-myelinating oligodendrocytes remain at calyx synapses. Finally, single cell electroporation determined developmental changes in oligodendrocyte process morphology. Together, our data suggest that oligodendrocytes mature and myelinate during the process of synaptic and overall circuit refinement in a region-specific manner.

## 2 Materials and methods

### 2.1 Animals

Mongolian gerbils (*Meriones unguiculatus*, based on Charles River hereditary background) between postnatal (P) day 5 and 59 and Etruscan shrews (*Suncus etruscus*) between 9 and 13 months of both sexes were included in this study. Animals were bred at the institute’s animal facility and kept under 12 h light/dark cycle. Gerbils had access to food and water *ad libitum* and Etruscan shrew husbandry corresponds to previously described conditions (Zacher and Felmy, 2024). Experiments were approved under the license number TiHo-2021-4, compliant with German local and federal laws. For estimating the sample size no power analysis was performed, because no data exists to support this calculation. The decision on sample size was made according to the estimated heterogeneity of the data sets.

### 2.2 Tissue preparation

**Figure 1:**
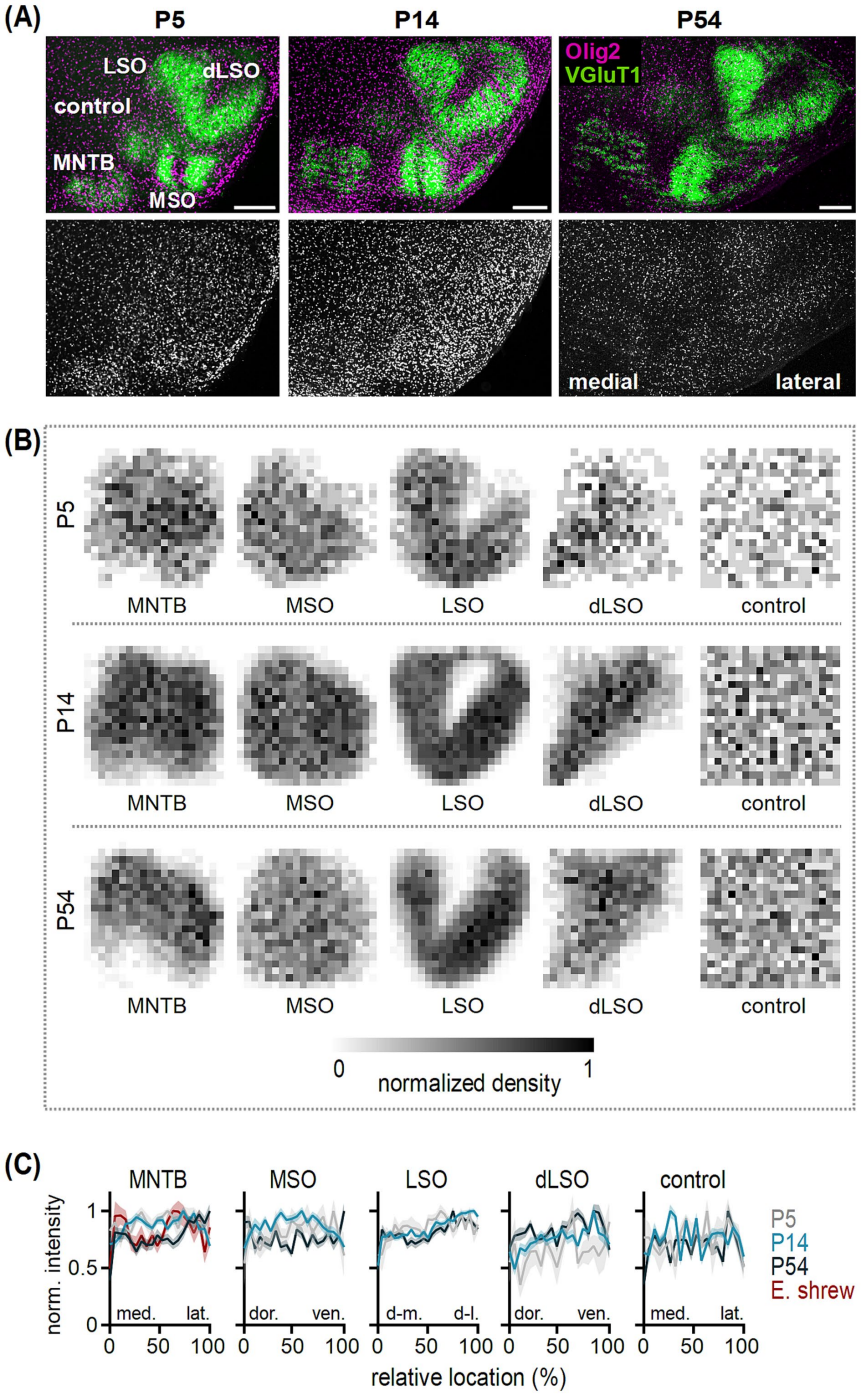
Oligodendrocyte distribution in the developing SOC nuclei. **(A)** Top: Olig2 (magenta) and VGluT1 (green) co-labeling in transversal brainstem sections from P5, P14 and P54 gerbils. VGluT1 labeling indicates the medial nucleus of the trapezoid body (MNTB), medial superior olive (MSO) and lateral superior olive (LSO). The VGluT1-negative dorsal LSO area (dLSO) represents the wedge between the two VGluT1-positive LSO limbs. The control region is located in a VGluT1-negative area dorsal to the MNTB. Scale bars equal 200 μm. Bottom: Olig2 labeling from images in the top row. **(B)** Distribution of oligodendrocytes in the MNTB, MSO, LSO, dLSO and control area in P5, P14 and P54 gerbils. Average normalized density of counted oligodendrocytes determined from single sections per nucleus and age group, visualized by normalized gray values. Number (*n*) of sections/animals/litters (age group): 5/3/2 (P5), 14/2/2 (P14), 18/6/3 (P50-59, grouped to P54). **(C)** Normalized intensity as a function of relative location at P5 (gray), P14 (light blue) and P54 (dark blue) obtained from normalized density plots of single sections [*n* for gerbil see **(B)**, *n* of sections/animals/litters for Etruscan shrew: 3/3/2]. Intensity profiles are shown in medio-lateral orientation for the MNTB and control region, dorso-ventral orientation for the MSO and dLSO and dorsomedial-dorsolateral orientation in the LSO. Lines show average values and shaded areas the SEM. Note that for the MNTB data from adult Etruscan shrews was added (red). Average intensities between 5 and 15% (medial, dorsal or dorsomedial) and 85–95% (lateral, ventral or dorsolateral) of the relative location were statistically compared. MNTB: gerbil P5 *p* = 0.923^U^, P14 *p* = 0.929^U^, P54 *p* = 0.003^U^; Etruscan shrew *p* = 0.415^M^. MSO: P5 *p* = 0.258^U^, P14 *p* = 0.689^U^, P54 *p* = 0.331^M^. LSO: P5 *p* = 0.055^M^, P14 *p* < 0.001^U^, P54 *p* = 0.051^M^. dLSO: P5 *p* = 0.414^M^, P14 *p* < 0.001^M^, P54 *p* = 0.003^M^. Control: P5 *p* = 0.938^U^, P14 *p* = 0.923^M^, P54 *p* = 0.704^M^. ^U^ = unpaired *t*-test, ^M^ = Mann Whitney U test.

**Figure 2:**
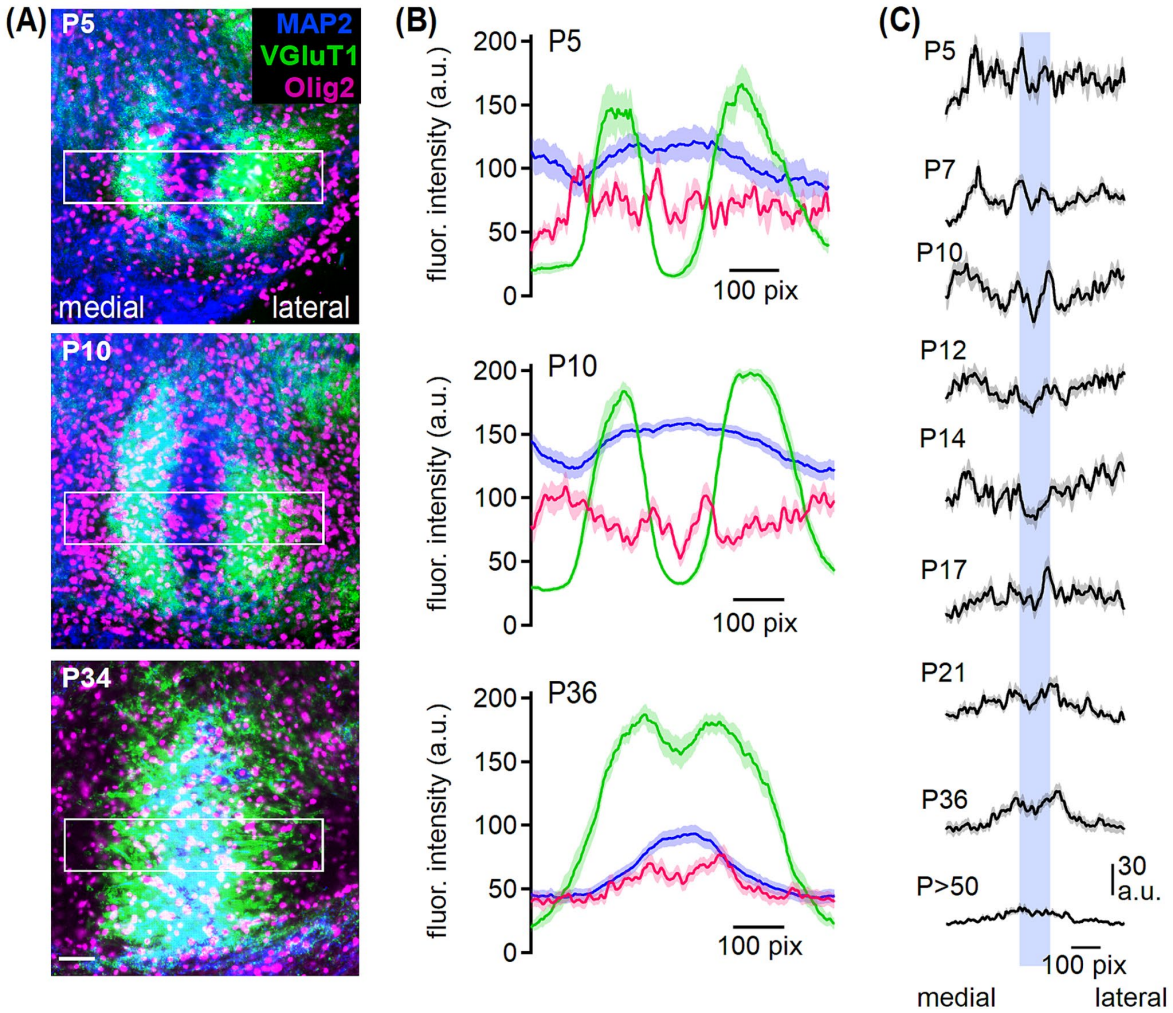
Oligodendrocyte arrangement in the developing MSO. **(A)** MAP2 (blue), VGluT1 (green) and Olig2 (magenta) co-labeling in the MSO from P5, P10 and P34 gerbil. Rectangles show the respective profile area of 359 × 72 μm. Scale bar equals 50 μm. **(B)** Overlaid fluorescence intensity profiles of arbitrary units (a.u.) of MAP2 (blue), VGluT1 (green) and Olig2 (magenta) at P5, P10 and P36. Medial to lateral from left to right. **(C)** Intensity profiles of Olig2 at the given postnatal ages. Blue bar represents the location of MSO somata based on MAP2 labeling. Number of sections/animals/litters (age group): 5/3/2 (P5), 23/4/2 (P7), 13/3/2 (P10), 16/4/2 (P12), 26/2/2 (P14), 26/3/2 (P17), 18/4/3 (P21), 22/4/4 (P34-38; grouped to P36), 18/6/3 (P54).

**Figure 3:**
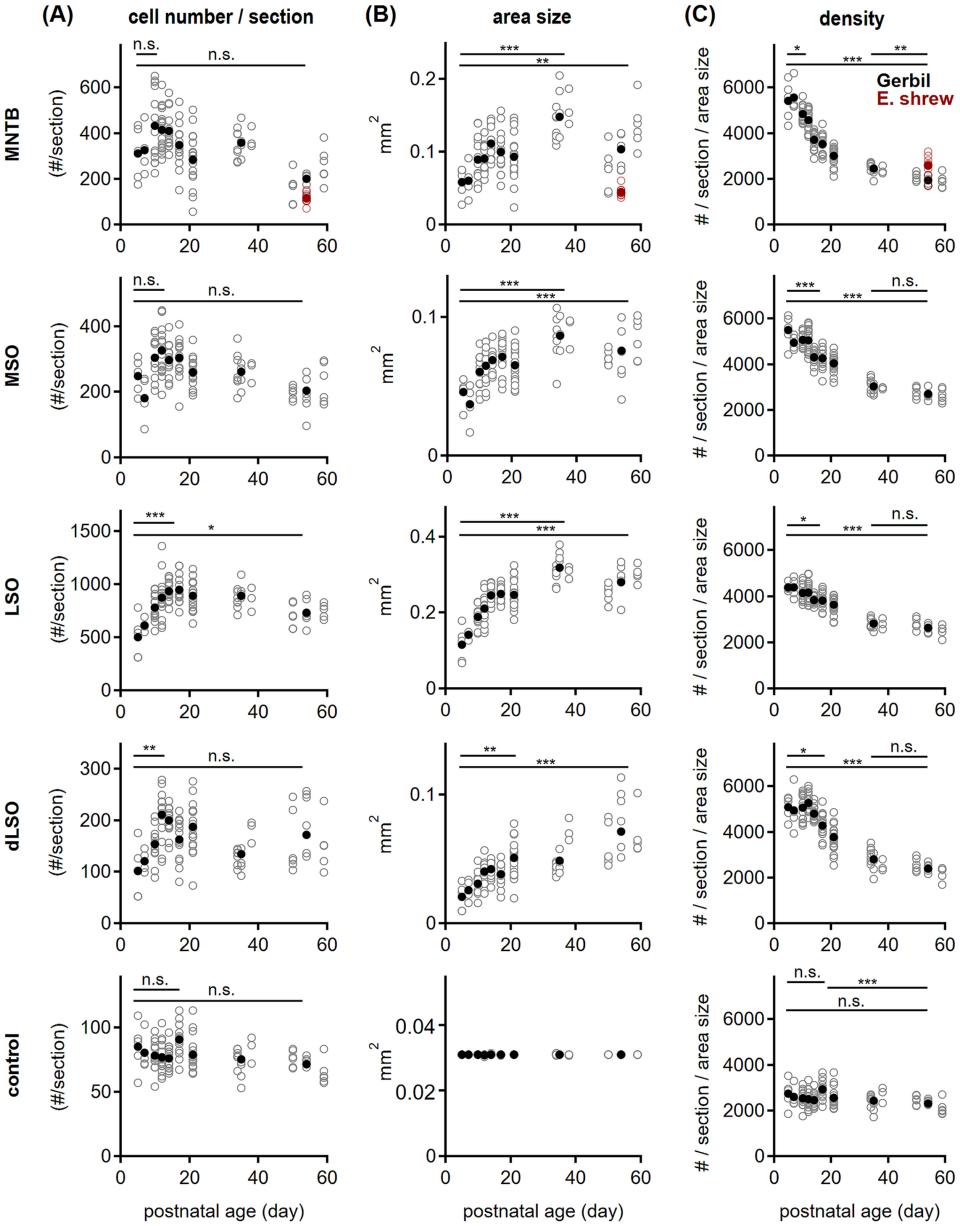
Development of oligodendrocyte quantity and density in the SOC. **(A)** Oligodendrocyte cell number per section in gerbil (black) plotted as a function of postnatal age for the given SOC nuclei: MNTB, MSO, LSO, dLSO and the control region (from top to bottom). For the MNTB, data from adult Etruscan shrew is shown (red). Open symbols represent values per section, filled symbols averages per age group. Comparison of P5 to peak: MNTB P5 vs. P10: *p* = 0.34^A^; MSO P5 vs. P12: *p* = 0.12^A^; LSO P5 vs. P14: *p* < 0.001^A^; dLSO P5 vs. P12: *p* = 0.01^K^; control P5 vs. P17 *p* > 0.99^K^. Comparison of P5 and P54: MNTB *p* = 0.34^A^, MSO *p* = 0.57^A^, LSO *p* = 0.02^A^, dLSO *p* = 0.51^K^; control *p* = 0.60^K^. Etruscan shrew vs. gerbil: *p* = 0.003^M^. ^A^ = One-way ANOVA with post-hoc Holm-Šídák’s test, ^K^ = Kruskal-Wallis with post-hoc Dunn’s test. Asterisks denote statistical differences between the age groups: not significant (n.s.) for *p* > 0.05, *p ≤ 0.05, **p ≤ 0.01, ***p ≤ 0.001. Number of sections/animals/litters (age group): 5/3/2 (P5), 4/2/1 (P7), 13/3/2 (P10), 16/3/2 (P12), 14/2/2 (P14), 14/3/2 (P17), 18/4/2 (P21), 15/3/3 (P36), 18/6/3 (P54), 3/3/2 (Etruscan shrew). **(B)** Same as in **(A)** but for the nucleus size (area size). Comparison between age groups: P5 vs. P36: *p* < 0.001^A^ for MNTB, MSO and LSO; P5 vs. P54: *p* = 0.01^A^ for MNTB, *p* < 0.001^A^ for MSO and LSO. dLSO P5 vs. P21 *p* = 0.004^A^, P5/P21 vs. 54: *p* < 0.001^A^. Comparison between SOC nuclei: P5 *p* = 0.002^K^, P54: *p* < 0.001^K^. Etruscan shrew vs. gerbil: *p* < 0.001^M^. Number of *n* as in **(A)**. **(C)** Same as in **(A)** but for oligodendrocyte cell density (in number/mm^2^). Comparison between age groups: MNTB: P5 vs. P10 *p* = 0.04^A^, P36 vs. P54 *p* = 0.004^A^, P5 vs. P54: *p* < 0.001^A^. MSO: P5 vs. P14 *p* < 0.001^A^, P36 vs. P54 *p* = 0.18^A^, P5 vs. P54: *p* < 0.001^A^. LSO: P5 vs. P14 *p* = 0.02^A^, P36 vs. P54 *p* = 0.71^A^, P5 vs. P54: *p* < 0.001^A^. dLSO: P5 vs. P17: *p* = 0.03^A^, P36 vs. P54 *p* = 0.25^A^, P5 vs. P54 *p* < 0.001^A^. Control: P5 vs. P17: *p* > 0.99^K^; P17 vs. P54: *p* < 0.001^K^; P5 vs. P54: *p* > 0.99^K^. Comparison between SOC nuclei: P5 *p* = 0.05^A^, P54: *p* < 0.001^A^. Etruscan shrew vs. gerbil: *p* < 0.001^M^. Number of *n* as in **(A)**.

**Figure 4:**
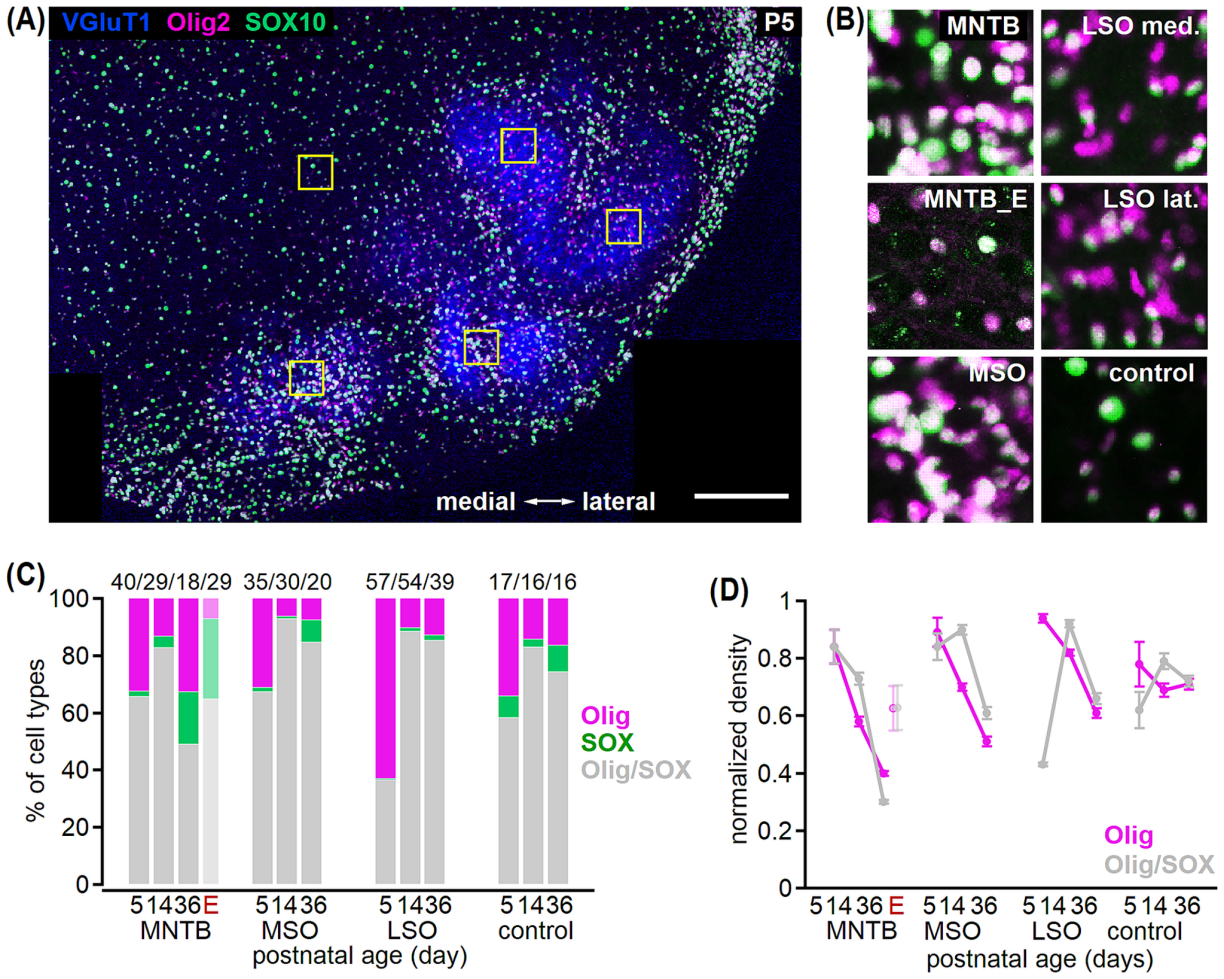
Olig2- and SOX10-based oligodendrocyte identification in the developing SOC. **(A)** Example co-labeling of VGluT1 (blue), Olig2 (magenta), and SOX10 (green) in a transversal brainstem section from a P5 gerbil. Squares show areas of cell counting comprising a 72 × 72 μm region in the MNTB, MSO, medial and lateral LSO as well as in the control region. Note that data from the LSO was pooled. Scale bar equals 200 μm. **(B)** Insets from the marked areas in **(A)** show Olig2 and SOX10 co-labeling in the MNTB, MSO, medial (med.) and lateral (lat.) LSO and control region. Additionally, co-labeling in the MNTB of mature Etruscan shrew (MNTB_E) is depicted. **(C)** Percentage of oligodendrocyte cells detected with Olig2 (magenta), SOX10 (green) or both (gray) markers at P5, P14, and P36 in the MNTB, MSO, LSO and control area in gerbil. For the MNTB, percentages from adult Etruscan shrew (E) are added. Total cell number is given above for each age group. Comparison of single SOX10-positive fraction between age groups: LSO *p* = 0.21^K^, MSO and MNTB *p* < 0.001^K^, control: *p* = 0.005^K^. Comparison of single Olig2-positive fraction before vs. after hearing onset: P5 vs. P14: MNTB^A^, MSO^K^, LSO^K^ *p* < 0.001, control^K^ *p* = 0.1. Number of sections/animals/litters (age group): 8/3/2 (P5), 16/2/1 (P14), 16/2/1 (P36), 3/3/2 (Etruscan shrew). **(D)** Normalized density of oligodendrocytes either detected by Olig2 (magenta) or Olig2/SOX10 co-labeling (gray) in gerbil. For the MNTB, data from adult Etruscan shrew (E) is added. Comparison of single Olig2 vs. Olig2/SOX10 double-labeled cells: P5: MNTB *p* > 0.99^A^, MSO *p* = 0.347^A^, LSO *p* < 0.001^K^, control *p* = 0.187^A^; P14: MNTB *p* = 0.143^A^, MSO *p* < 0.001^A^, LSO *p* = 0.166^K^, control *p* = 0.976^A^. Number of *n* as in **(C)**.

**Figure 5:**
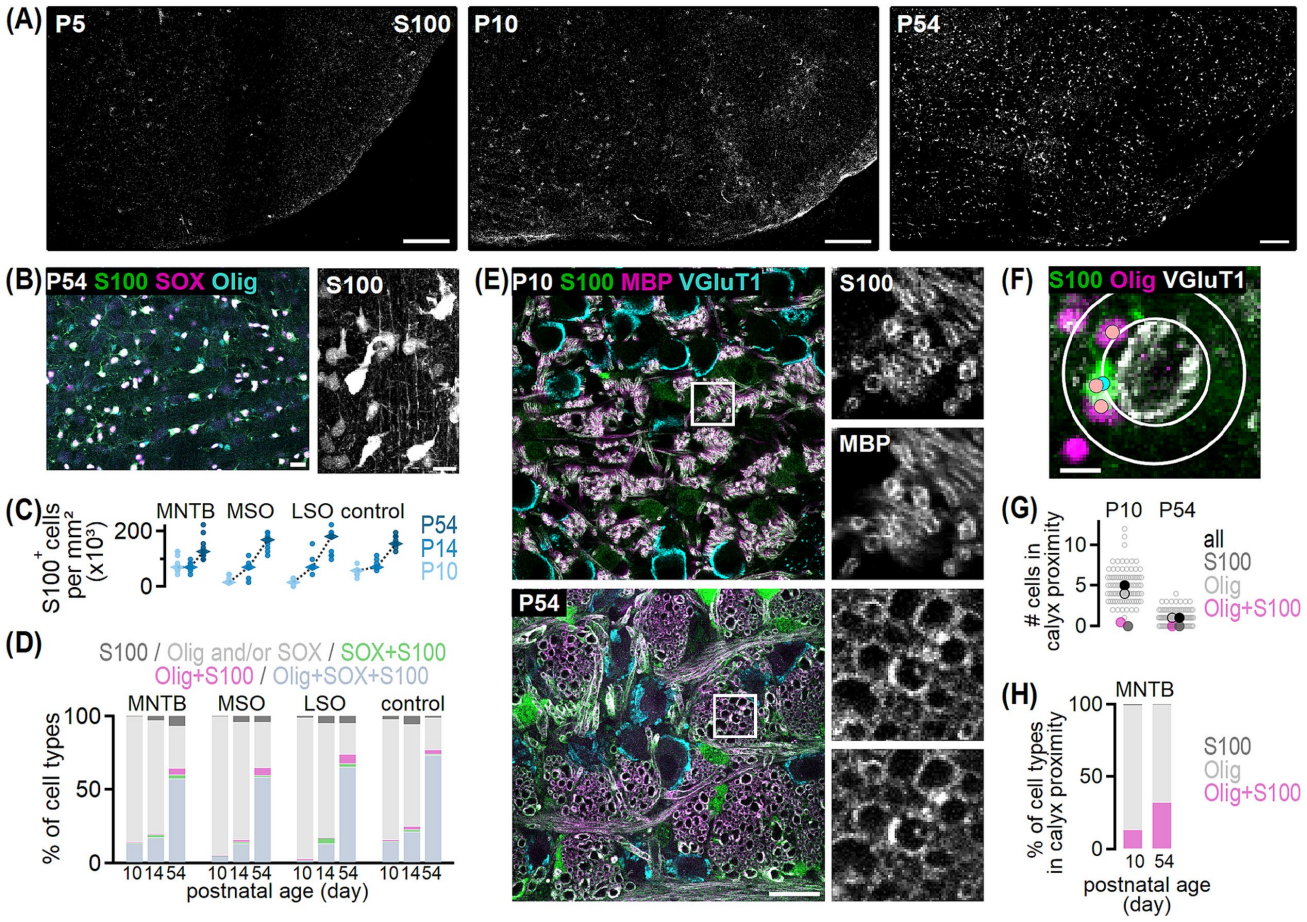
S100 labeled oligodendrocytes in the developing SOC. **(A)** Example overviews of S100 labeling in transversal SOC sections from P5, P10 and P54 gerbil. Scale bars equal 150 μm. **(B)** Left: S100 (green) co-labeling with SOX10 (magenta) and Olig2 (cyan) in the gerbil MNTB at P54. Right: Maximum projection showing S100-positive cells from image on the left at higher magnification. Scale bars equal 20 μm (left) and 15 μm (right). **(C)** Cell density of S100-positive cells in the four given regions at P10, P14, and P54. Dots show values per section and rhombs median densities. Comparison between regions: P10 *p* < 0.001^K^, P14 *p* = 0.678^A^, P54 *p* = 0.552^A^ and age groups: P10 vs. P14: MNTB, control *p* > 0.999^K^, MSO *p* = 0.689^K^, LSO *p* = 0.017^K^; P10 vs. P54 and P14 vs. P54: *p* < 0.001^A^. Number of sections/animals/litters per age group: 3/3/2. **(D)** Percentage of cells detected with S100, Olig2 and/or SOX10, SOX10 and S100, Olig2 and S100 or Olig2/SOX10/S100 triple labeling in the SOC and control region at P10, P14, and P54. Number of *n* as in **(C)**. **(E)** Example overviews of S100 (green), MBP (magenta), and VGluT1 (cyan) co-labeling in sagittal sections of the MNTB at P10 and P54. Squares mark regions of defined size (20 × 20 μm) where S100/MBP single and double labeled axons were counted. Insets show S100 and MBP labeling in the marked area at higher magnification. **(F)** Methodology to quantify the number of S100 (green) and Olig2 (magenta) single and double labeled cells in proximity of VGluT1-positive calyx-MNTB neuron synapses (gray). The sampling area where cells were considered in calyx proximity (outer white circle), was determined for each cell individually using the sum of the circle diameter surrounding a given calyx (inner white circle) and the average diameter of a S100-positive cell. Cells were counted and assigned as S100 (blue dot) or Olig2 single labeled (pink dot) or double labeled cells (blue and pink dot). Scale bar equals 10 μm. **(G)** Number of oligodendrocytes in calyx proximity at P10 and P54. Counts per calyx are shown by unfilled dots and represent the respective sum of all cell types. Filled dots show average values of all oligodendrocyte types (black), single S100- (dark gray) or Olig2-positive (light gray framed) and double labeled (magenta) cells. Number of sections/animals/litters/calyces (age group): 11/2/1/88 (P10), 11/2/1/89 (P54). **(H)** Average percentage of oligodendrocyte cell types in calyx proximity at P10 and P54. Number of *n* as in **(G)**.

**Figure 6:**
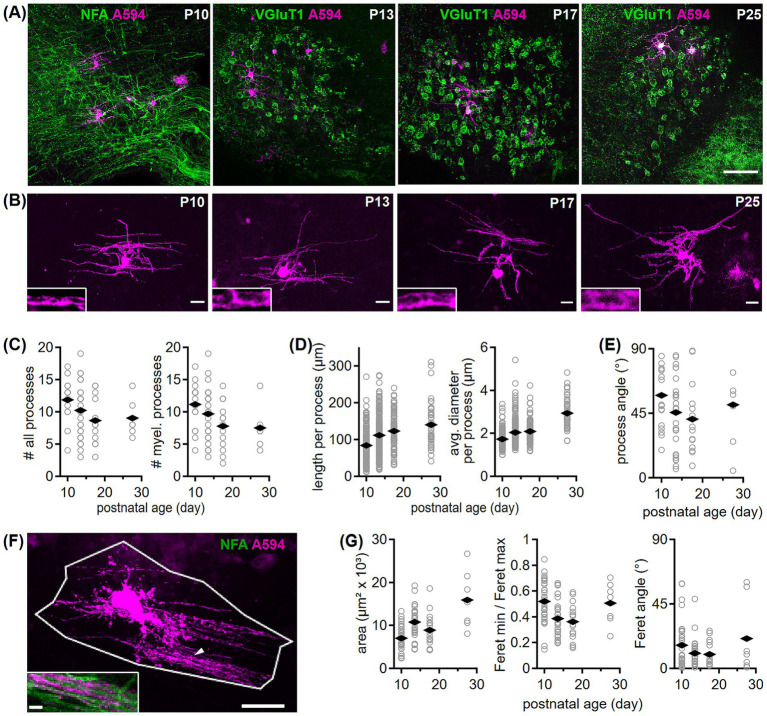
Development of oligodendrocyte morphology in the gerbil MNTB. **(A)** Example overviews for the four tested age groups showing single labeled oligodendrocytes (Alexa594, magenta) in the MNTB. Acute sections containing electroporated oligodendrocytes were fixed and post-hoc immunofluorescently labeled with NFA or VGluT1 (green). Scale bar equals 100 μm. **(B)** Maximum projection images of confocal stacks taken from single labeled oligodendrocytes shown in **(A)**. Scale bars equal 20 μm. Insets of 8 × 3.2 μm (maximum projection) captured at higher magnification from oligodendrocyte processes at the specified age groups, display intensity profiles that likely represent axonal myelination. **(C)** Number of all processes per oligodendrocyte containing non-myelinating blind endings (left, P10 vs. >P25: *p* = 0.106^U^) and number of putatively myelinating (myel., P10 vs. > P25: *p* = 0.04^U^) processes per cell (right) in four age groups. Open symbols represent individual cells and rhombs averages per age group. Number of cells/animals/litters (age group): 17/3/3 (P10), 27/7/6 (P13/14), 14/5/5 (P17/18), 7/5/5 (P25-30). **(D)** Length (P10 vs. P13/P17/<P25: *p* < 0.001^K^; P13 to P17: *p* = 0.07^K^) and diameter (P13/P17/<P25: *p* < 0.001^K^; P13 to P17: *p* = 0.36^K^) of oligodendrocyte processes. Symbols and number of *n* as in **(C)**. **(E)** Orientation of oligodendrocyte processes given as angle to the medio-lateral axis. Comparison between age groups: *p* = 0.99^A^. Symbols and number of *n* as in **(C)**. **(F)** Maximum projection of a P10 oligodendrocyte (A594, magenta) framed by a polygon exemplifies coverage area determination. Polygon boundaries were based on the most outer processes. Inset shows labeled oligodendrocyte processes (marked by arrowhead) together with NFA (green) labeling at higher magnification. Scale bars equal 25 μm (overview), 5 μm (inset). **(G)** Coverage area (P10 vs. >P25: *p* < 0.001^A^; P13 vs. P17: *p* = 0.19^A^), area shape (ratio of minimum to maximum Feret; comparison between age groups: *p* < 0.001^A^) and the area’s Feret angle (comparison between age groups: *p* = 0.68^K^) for the four age groups. Symbols and number of *n* as in **(C)**.

To prepare fixed sections for cell counting (Figures 1–5), animals were euthanized by inhalation of carbon dioxide and declared dead after at least 1–2 min breathing arrest. Subsequently, they were transcardially perfused with Ringer solution containing heparin, followed by 2% paraformaldehyde (PFA) containing 15% picric acid for fixation. Brains were removed, post-fixed overnight at 4°C in PFA and washed with phosphate-buffered saline (PBS) consisting of (in mM): 138 NaCl, 2.7 KCl, 10.2 Na_2_HPO_4_, and 1.76 KH_2_PO_4_, adjusted to pH 7.4. Transversal (Figures 1–5) or sagittal (Figure 5F) sections of 50 μm were prepared using a Ci 7000 vibratome (Campden Instruments) and stored in PBS. To prepare acute slices for single cell electroporation (Figure 6), gerbils were deeply anaesthetized with isoflurane, decapitated and the brains were rapidly removed in ice-cold NMDG solution consisting of (in mM): 30 NaHCO₃, 1.2 NaH₂PO₄, 2.5 KCl, 25 Glucose, 20 Hepes, 93 NMDG, 10 MgCl₂, 2 CaCl₂, 93 HCl, 0.49 L-Ascorbic acid, 0.3 Myo-inositol, 0.29 Na-pyruvate, bubbled with 95% O₂ and 5% CO₂ resulting in a pH of 7.4. Transversal sections of 200 μm were prepared using the Leica VT1200S vibratome, then incubated at 37°C for 7 min in NMDG solution bubbled with 95% O₂ and 5% CO₂, and subsequently stored at room temperature in recording solution containing (in mM): 125 NaCl, 25 NaHCO₃, 2.5 KCl, 1.25 NaH₂PO₄, 1 MgCl₂, 1.2 CaCl₂, 25 glucose, 0.4 ascorbic acid, 3 myo-inositol, and 2 Na-pyruvate, bubbled with 95% O₂ and 5% CO₂ with a pH of 7.4.

### 2.3 Electroporation

For detailed morphological analysis, oligodendrocytes were electroporated in acute brain sections following a procedure similar to that described previously (Wicke et al., 2023). During electroporation, brain sections containing the SOC were perfused continuously with recording solution at 34–36°C. Electroporation was accomplished under visual control of a CCD camera (T.I.L.L.Photonics GmbH) mounted onto an upright microscope (BX50I, OLYMPUS) with a 40×/0.80 W objective. Cell loading was monitored with a monochromator system (T.I.L.L.Photonics GmbH). First, the MNTB region was searched. In initial trial experiments, all cells with somas smaller than MNTB neurons were electroporated, resulting in the labeling of astrocytes, microglia and oligodendrocytes. Because electroporated cells were directly visualized, it was observed that oligodendrocytes exhibited larger cell bodies than astrocytes and microglia, with a distinct oval shape. Additionally, oligodendrocytes were often located at axonal fiber tracts and in close proximity to cells of similar size and shape. The later observation aligns with the immunofluorescence labeling, where oligodendrocytes were observed in clustered accumulations (Figures 1–4). These features allowed sufficiently for an identification of oligodendrocytes during the electroporation procedure. Visually identified oligodendrocytes within the MNTB were approached with a glass pipette (3.5–5 MΩ) filled with Alexa Fluor 594 sodium hydrazide (10 mM). The dye-loaded pipette was pressed onto the selected cell and a single voltage pulse was applied (10–15 ms, 10–15 mV). The voltage pulse was generated by an EPC10 amplifier with Patchmaster software (HEKA Elektronik) and amplified by a stimulus isolator (A-M Systems Model 2100). Sections containing the loaded cells were fixed in 4% PFA for 3 h at room temperature or overnight at 4°C. Thereafter, the sections were washed three times and stored in PBS for subsequent immunofluorescence labeling.

### 2.4 Immunofluorescence labeling

Free floating sections for cell counting were washed with sodium borohydride (1 mg/mL, diluted in PBS) four times for 10 min on ice and thereafter with blocking solution, containing 0.5% Triton, 1% bovine serum albumin and 0.1% saponin (Roth, Karlsruhe, Germany) diluted in PBS, for 30 min at room temperature. Sections were then incubated for 3 days at 4°C in blocking solution containing the primary antibodies: microtubule-associated protein 2 (MAP2), chicken, 1:1000, Origene (Herford, Germany), Cat# TA336617; myelin basic protein (MBP), rat, 1:500, Abcam (Cambridge, UK), Cat# ab7349, RRID:AB_305869; Olig2, rabbit, 1:3000, ProteinTech (Planegg-Martinsried, Germany), Cat# 13999-1-AP, RRID:AB_2157541; SOX10, goat, 1:1000, R&D Systems (Wiesbaden, Germany), Cat# AF2864, RRID:AB_442208; S100, mouse, 1:5000, Thermofisher (Darmstadt, Germany), Cat# MA5-12969, RRID:AB_10983883; vesicular glutamate transporter 1 (VGluT1), guinea pig, 1:2000, Synaptic Systems (Goettingen, Germany), Cat# 135304, RRID:AB_887878. Subsequently, sections were washed in washing solution, containing 0.5% Triton and 0.1% saponin diluted in PBS, and then incubated for 4 h at room temperature in blocking solution containing the secondary antibodies: AMCA, anti-chicken, 1:100, Cat# 703-156-155, RRID:AB_2340362; Cy3, anti-rat, 1:200, Cat# 712-165-153, RRID:AB_2340667; Cy3, anti-rabbit, 1:800, Cat# 711-165-152, RRID: AB_2307443; Alexa488, anti-rabbit, 1:800, Cat# 711-545-152, RRID:AB_2313584; Cy3, anti-goat, 1:800, Cat# 705-165-147, RRID: AB_2307351; Alexa488, anti-mouse, 1:200, Cat# 715-545-150, RRID:AB_2340846; Alexa488, anti-guinea pig, 1:400, Cat# 703-546-148, RRID: AB_2340473. All secondary antibodies were purchased from Dianova (Hamburg, Germany). Sections were then washed in washing solution and mounted on gelatinized microscope slides using fluorescence mounting medium (VECTASHIELD©, Vector Laboratories).

Fixed sections containing electroporated cells were washed with blocking solution for 30 min at room temperature, incubated for 2–3 days at 4°C with blocking solution containing the primary antibody (VGluT1, guinea pig, 1:2000, Synaptic Systems, Cat# 135304, RRID: AB_887878 or neurofilament-associated antigen (NFA), mouse, 1:500, DSHB (Iowa, United States), Cat# 3A10, RRID: AB_531874) and subsequently washed three times before incubation overnight at 4°C with the secondary antibody (Alexa488, donkey anti-guinea pig, 1:400, Cat# 703–546-148, RRID: AB_2340473 or Alexa488, donkey anti-mouse, 1:200, Cat# 715–545-150, RRID: AB_2340846) diluted in blocking solution. Sections were then washed once with blocking solution and three times with PBS each for 10 min, and then mounted as described above. Unless specified otherwise, all chemicals were obtained from Sigma-Aldrich (Darmstadt, Germany).

### 2.5 Antibody specificity

The MAP2 antibody is made against AA 377-1505 of recombinant human MAP2 in chicken. According to the manufacturer’s datasheet, this antibody recognizes a single band of 280 kDa on Western blot of bovine brain. MAP2 labels the soma and dendrites of neurons in all major SOC nuclei of mice (Rajaram et al., 2019), the MSO of gerbil (Callan et al., 2021) and the MNTB in bats (Kladisios et al., 2023). The MBP antiserum is made against AA 82–87 of recombinant MBP in rat and recognizes the appropriate bands on Western blots of rat and mouse brain (manufacturer’s datasheet). This antibody reliably labels myelin in the MNTB of mouse (Sinclair et al., 2017). The Olig2 antibody was purified in rabbit and recognizes the appropriate bands on Western blots of rat and mouse brain (manufacturer’s datasheet). At least in mice and macaques this antibody labels oligodendrocyte somata (Yao et al., 2022; Martin et al., 2022). The SOX10 antiserum was raised in goat and detects human SOX10 in direct ELISAs and Western blots (manufacturer’s datasheet). It is a standard antibody to detect oligodendrocytes at least in mice (Kohnke et al., 2021; Khandker et al., 2022; Jeffries et al., 2021; Buller et al., 2023; Bai et al., 2023). The S100 antibody is made against AA 1-92 of purified bovine brain S100 protein in goat and shows a 9 kDa band in Western blot of mouse brain (manufacturer’s datasheet). S100 labels oligodendrocytes in various brain regions at least in rat (Ludwin et al., 1976; Rickmann and Wolff, 1995), cat (Dyck et al., 1993), and mouse (Du et al., 2021). For the VGluT1 antiserum, which is made against AA 456-560 of recombinant VGluT1 in rat, we used a higher dilution than suggested by the manufacturer. This antibody recognizes the appropriate bands on Western blots of rat and mouse brain, and no band was seen in VGluT1 knock-out mice (manufacturer’s datasheet). VGluT1 labels excitatory synaptic inputs in all major SOC nuclei of mice (Fischl et al., 2016), gerbil (Zacher et al., 2023), bat (Pätz et al., 2022), and Etruscan shrew (Zacher and Felmy, 2024). The NFA antibody, made in mouse, recognizes a neurofilament-associated antigen and reliably labels axons in zebrafish (Mathews et al., 2014), frog (Santos et al., 2022), rat (Panlilio et al., 2023), and mouse (Frendo et al., 2019).

### 2.6 Image acquisition

Acquisition of overview images for cell counting of Olig2- (gerbil, Etruscan shrew) and Olig2/SOX10- (gerbil) positive cells (Figures 1–4) was controlled by IC capture software version 2.4 (The Imaging Source Europe GmbH, Bremen, Germany) and performed using a Zeiss Axioplan2 (Zeiss, Goettingen, Germany) with a 10×/0.3 NA objective equipped with a USB CMOS camera (DFK 23UX249, The Imaging Source Europe GmbH), leading to a pixel size of 1.67 μm. AMCA, Alexa488 and Cy3 fluorescence was captured with excitation (360/50, 470/40, and 546 nm), dichroic (400, 495, and 580 nm) and emission (460/50, 525/50, and 590 nm) filters, respectively. To image the entire SOC and our defined dorsal control region, tile images within a given section were taken with the same acquisition settings. Color channels were post-hoc combined in Fiji (Schindelin et al., 2012) and tile images were merged using the stitching plugin (Preibisch et al., 2009). Olig2/SOX10 co-labeling in Etruscan shrews was imaged with an inverted confocal microscope (Leica TCS SP5, Leica microsystems, Wetzlar, Germany) equipped with an argon, 561 and 633 nm laser to detect Alexa488, Cy3 and Alexa 647, respectively. Images containing the MNTB were acquired with a 63×/1.40 NA oil immersive objective and a pixel size of 0.246 μm. S100/Olig2/SOX10 triple labeling was imaged with the same confocal system. Z-stacks were taken with 10 consecutive images per region (MNTB, MSO, LSO and control area) using a 20×/0.7 NA objective, 2× optical zoom and a step size of 0.42 μm yielding a voxel size of 0.379 μm × 0.379 μm × 0.42 μm. For the S100/MBP/VGluT1 triple labeling, three consecutive images were acquired from the MNTB using a 40×/1.25 NA oil immersive objective, 2.5× optical zoom and a step size of 0.29 μm yielding a voxel size of 0.303 μm × 0.303 μm × 0.29 μm. Maximum projection images were generated from all z-stacks in Fiji and used for further analysis. For the S100/Olig2/VGluT1 triple labeling, stacks with 0.712 μm z-step size were acquired from the MNTB using a 20×/0.7 NA oil immersive objective and 2× optical zoom yielding a voxel size of 0.758 μm × 0.758 μm × 0.712 μm.

Images for detailed morphological analysis were acquired with a Leica TCS SP5 and TCS SP8 (Leica microsystems). Sections were always oriented in such a way that the midline aligned vertically. For image acquisition with the Leica TCS SP5, equipped with an argon and 594 nm laser to detect Alexa488 and Alexa594, respectively, samples were magnified by a 40×/1.25 NA oil immersive objective and additional 1.5× optical zoom. Z-stacks were taken with a step size of 0.52 μm yielding a voxel size of 0.505 μm × 0.505 μm × 0.502 μm. Imaging with the Leica TCS SP8 equipped with an argon laser and DPSS 561 nm-laser to detect Alexa488 and Alexa594, respectively, was performed with a 20×/0.5 NA oil immersion objective and 2× optical zoom. Sampling with 0.55 μm z-step size yielded a voxel size of 0.505 μm × 0.505 μm × 0.505 μm. With both systems, acquisition of tile images was required to obtain full oligodendrocytes with their partially long processes. The gain was thereby manually adjusted over a full image throughout scanning to depict fine cell processes. Color channels were merged post-hoc in Fiji. In case tile images were acquired for a given cell, files were combined using the Fiji stitching tool. Stacks for example images showing myelinating processes (Figure 6B) were taken at higher magnification using a 63 × 1.4NA oil immersion objective and 3× optical zoom yielding a voxel size of 0.04 μm × 0.04 μm × 0.04 μm.

### 2.7 Data analysis

#### 2.7.1 Cell counts and distribution

To quantify the number of oligodendrocytes within SOC nuclei, nuclear boundaries of the MNTB, MSO and LSO were drawn based on VGluT1 labeling using the freehand tool in Fiji. A VGluT1-negative dorsal LSO region (dLSO) was drawn between the two VGluT1-positive LSO limbs and a control region (300 × 300 pixel corresponding to 179 × 179 μm) was assigned to a VGluT1-negative area dorsal of the MNTB. With the point tool, each Olig2-positive oligodendrocyte soma center was marked within the assigned regions. Image brightness of the Olig2 channel was adjusted from zero to maximum intensity to detect faint cells located in the deeper tissue. With a custom written Fiji macro, the area (nucleus size) and the centroid position with inverted Y coordinates (cell position) was extracted and exported as csv file for further analysis. The csv files were processed in R Studio to analyze nucleus area, cell location, cell number and cell density within the marked nuclear boundaries. Here, the following number of sections, animals and litters were included [sections/animals/litters (age group)]: 5/3/2 (P5), 4/2/1 (P7), 13/3/2 (P10), 16/3/2 (P12), 14/2/2 (P14), 14/3/2 (P17), 18/4/2 (P21), 15/3/3 (P34-38; grouped to P36), 18/6/3 (P50-59, grouped to P54).

To analyze the distribution of counted oligodendrocytes, the relative abundance of cells was determined per nucleus and age group using a custom written R script. First, we standardized the respective nucleus size per section by setting the ventro-dorsal (Y-axis) and medio-lateral (X-axis) dimensions to 100%. Then, we divided these distances into 5% increments. The highest cell count in a nucleus of a single section was used to normalize the cell density for this section. The normalized density, which ranges from zero to one, was then calculated for each 5% increment and visualized using normalized gray values. A higher abundance of cells appeared as darker gray tone. Data from single sections were averaged for each nucleus and age group along the 5% increments. Intensity profiles were generated from single sections using IgorPro (Version 9.02, Wavemetrics, Portland, United States). Profiles were centered at 50% ± 10% relative location and were taken with 50% total width in medio-lateral orientation for the MNTB and control region, and dorso-ventral orientation for the MSO. Because periolivary neurons in the dLSO appear not tonotopically arranged (Williams et al., 2022), the longest axis, and, thus, a dorso-ventral orientation was chosen. For the LSO, profiles were determined freehand to adapt to the LSO shape in dorsomedial-to-ventral-to-dorsolateral orientation and covered 30% of total width at each position. Therefore, line scan directions are attributed to the anticipated tonotopy in the SOC nuclei (Kandler et al., 2009).

For the comparison of Olig2 and SOX10 labeling, Olig2- and SOX10-positive cells were counted in the MNTB, MSO, medial and lateral limb of LSO and a control region using a 120 × 120 pixel rectangle corresponding to 72 × 72 μm. Cell counts for the medial and lateral LSO limb were pooled because no differences were detected. Counting was conducted as described above with Olig2 and SOX10 labeling considered separately. Then, the counts were overlaid to determine the number of double-labeled somata. Here, the following number of sections, animals and litters were included: [sections/animals/litters (age group)]: 8/3/2 (P5), 16/2/1 (P14), 16/2/1 (P34-38; grouped to P36). In Etruscan shrews, Olig2- and SOX10-positive cells were counted similarly but in the entire MNTB. Therefore, the nuclear boundary was drawn based on VGluT1 labeling and nucleus size as well as cell position was extracted as described before to determine cell number and cell density within the nuclear boundary. For this, three adult animals from two litters with each three sections per animal were included.

To quantify S100 co-labeling with Olig2 and SOX10, the same procedure was applied in the four given regions with a rectangle of 190 × 190 pixel corresponding to 72 × 72 μm. MBP and S100 co-labeling was compared only in the MNTB within a region of 67 × 67 pixel corresponding to 20 × 20 μm. There, single S100 and MBP-positive as well as double labeled axons were counted. For both S100 co-labelings, three animals from two litters with each three sections per animal were included per age group.

To determine the amount of S100 and Olig2 single and double labeled cells in proximity of calyx-MNTB neuron synapses at P10 and P54, maximum projections of three to five consecutive images containing a given VGluT1-positive calyx synapse were used. Applying the oval tool in Fiji, a circle was drawn around the VGluT1-positive calyx and the circle diameter was extracted to calculate the sampling area where cells were considered in calyx proximity (Figure 5F). Therefore, the circle diameter and the average diameter of a S100-positive cell (17 μm, *n* = 68, P10) were summed and a second circle of the determined sampling area size was drawn centered in the center of the first circle. Cells having their cell bodies fully within the sampling area were counted with regard to their labeling as S100 single labeled, Olig2 single labeled or double labeled cells. For this analysis, the following number of sections, animals, litters and calyces were included: [sections/animals/litters/calyces (age group)]: 11/2/1/88 (P10), 11/2/1/89 (P54).

#### 2.7.2 Fluorescence intensity profiles

For MSO line scans, a rectangle (600 × 120 pixel corresponding to 359 × 72 μm) was placed centered on the MSO based on VGluT1 and MAP2 labeling. Fluorescence intensity profiles were obtained for Olig2, VGluT1 and MAP2 labeling using the ‘Plot Profile’ plugin in Fiji. Data from the left hemisphere was inverted so that always a medial-to-lateral alignment was given. For this analysis, we included the following number of sections from the given number of animals and litters [sections/animals /litters (age group)]: 5/3/2 (P5), 23/4/2 (P7), 13/3/2 (P10), 16/4/2 (P12), 26/2/2 (P14), 26/3/2 (P17), 18/4/2 (P21), 22/4/4 (P34-38; grouped to P36), 18/6/3 (P50-59, grouped to P54).

#### 2.7.3 Morphology

For this analysis, only oligodendrocytes located within the MNTB, a structure readily distinguishable by ring like VGluT1-positive structures reminiscent of the calyx of Held synapses, were used. Maximum intensity projections were used as overviews and z-stacks were utilized to allocate and track cell processes. First, the number of primary processes, here defined as processes directly emerging from the soma, their length and orientation (angle) to the medio-lateral axis were determined. Further, the process diameter was analyzed. Therefore, the length of a given process was divided by five, the diameter was measured at the center of each instance using the line tool and the average diameter per process was calculated. If process overlap existed at the center, the diameter was measured at sites where processes could be distinguished from one another. Moreover, the number of secondary processes, defined as processes emerging from primary processes, their length, orientation and diameter were measured. The remaining processes were counted as “blind endings,” which comprised processes ending abruptly after emanating from the cell body or outgrowths where myelination of axons was not detected. To verify that myelinating oligodendrocytes have been electroporated, three intensity profiles per cell were taken from 5.55 × 5.55 μm regions on different processes in one plane. A low center intensity flanked by two high intensity peaks accounted for myelination around an axon. For the analysis of process characteristics, we included the following number of cells from the given number of animals and litters [cells/animals/litters (age group)]: 17/3/3 (P10), 27/7/6 (P13/14), 14/5/5 (P17/18) and 7/5/5 (P25-30).

To assess how much tissue area can be covered by oligodendrocyte processes, the maximum projection images of the following number of cells from the given amount of animals and litters were used [cells/animals /litters (age group)]: 34/3/3 (P10), 31/7/6 (P13/14), 16/5/5 (P17/18), 7/5/5 (P25-30). Therefore, oligodendrocytes were bordered based on the most outer processes using the polygon tool and the area, minimum Feret, maximum Feret and Feret angle were extracted.

### 2.8 Statistical analysis

Data were visualized using IgorPro (Version 9.02, Wavemetrics) and statistical comparison was performed with Graphpad Prism 9.5.1 (Dotmatics, Boston, United states). Data were tested for normal distribution using the Shapiro–Wilk test. Not normal distributed data were given as median ± SEM and were compared using the Mann–Whitney U test (two groups) or Kruskal-Wallis with *post-hoc* Dunn’s test for multiple comparison (>two groups). Normal distributed data were given as mean ± SEM and were compared using an unpaired t-test (two groups) or one-way ANOVA with post-hoc Holm-Šídák’s multiple comparisons test (>two groups). A probability of *p* < 0.05 was considered statistically significant.

## 3 Results

### 3.1 Oligodendrocyte spatial and developmental redistribution

The distribution and density of Olig2 labeling, altered in the developing SOC (Figure 1). To localize Olig2-positive cells, further referred to as oligodendrocytes, within SOC nuclei, VGluT1 co-labeling was used as structural marker (Zacher et al., 2023). Here, in gerbil we focused on the medial nucleus of the trapezoid body (MNTB), medial superior olive (MSO) and lateral superior olive (LSO). Moreover, a VGluT1-negative dorsal LSO region (dLSO) and a VGluT1-negative control region outside of the SOC were examined. The dLSO was included because its periolivary neurons are an important component of the ascending auditory pathway and might form a distinct intrinsic circuitry (Williams et al., 2022; Adams, 1979; Schofield and Cant, 1992). This allowed comparison of oligodendrocyte arrangement between olivary, periolivary and a control region to address developmental rearrangement specificity.

We observed that the number of oligodendrocytes increased during the first two postnatal weeks and decreased thereafter (Figure 1A), and that their local distributions changed. To compare the distribution within nuclei and the control area across age groups, the average normalized density of counted oligodendrocyte cell bodies within the normalized nucleus area was determined at P5, P14, and P54 (Figure 1B). A lower cell density appeared at the border area of the nuclei compared to their inner region throughout development. Oligodendrocyte density appeared larger in SOC nuclei compared to the control region and local heterogeneities were apparent. In the MSO for example, a lower cell density was observed in the center compared to the medial and lateral region at P14 (Figure 1B).

For deeper analysis of the spatial distribution of oligodendrocytes, intensity profiles based on the normalized density plots were generated from single sections and averaged per nucleus and age group (Figure 1C). To account for the assumed tonotopy in the SOC nuclei (Kandler et al., 2009), profiles were taken in medio-lateral (MNTB, control), dorso-ventral (MSO, dLSO) or dorsomedial-dorsolateral orientation (LSO) and average intensities between 5 and 15% (medial, dorsal or dorsomedial) and 85–95% (lateral, ventral or dorsolateral) of the relative location were compared. In the MNTB, similar normalized intensities were detected independent of location at P5 and P14, while at P54 a higher normalized intensity and, thus, more oligodendrocytes were found in the lateral compared to the medial MNTB. In contrast to the gerbil MNTB, no differences between the medial and lateral MNTB were found in adult Etruscan shrew. In the MSO, no differences in intensity were detected between the dorsal and ventral region throughout development. For the LSO, no significant differences in intensity values between the dorsomedial and dorsolateral region were detected at P5 and P54, although an increasing trend toward the dorsolateral region was apparent reaching significance only at P14. In the dLSO, differences in intensity established after P5 showing higher values in the ventral compared to the dorsal region at P14 and P54. In the control region, similar intensity values appeared along the medio-lateral axis suggesting a homogenous oligodendrocyte distribution in non-nuclei regions throughout development. Hence, in the MNTB and LSO spatial rearrangements occurred along their assumed tonotopic axis with accumulations in the lateral, low frequency region (Kandler et al., 2009).

To confirm that during the second postnatal week fewer oligodendrocytes are located in the MSO center (Figure 1B), fluorescence intensity profiles of MAP2, VGluT1, and Olig2 co-labeling were analyzed in a medio-lateral alignment (Figure 2). MAP2 labels somata and dendrites of MSO neurons (Callan et al., 2021; Rautenberg et al., 2009). In young age groups (P5 and P10 exemplarily, Figure 2A), VGluT1 labeling showed a strong dendritic location with a negative band in the somatic area. Hence, glutamatergic inputs first localize to the dendritic neuropil. In the mature MSO, the VGluT1 fluorescence covered also the somatic area (Figures 2A,B) consistent with excitatory input refinement (Franzen et al., 2020). Therefore, we could use MAP2 and VGluT1 labeling as positional marker for the somatic and dendritic region, respectively. Olig2 labeling intensity indicated a homogeneous cell distribution at P5 (Figures 2B,C). At P10, oligodendrocytes accumulated at the proximal somato-dendritic and distal dendritic borders indicated by VGluT1 and hardly any cells appeared in the VGluT1-negative central somatic MSO region (Figure 2A). The Olig2 intensity profile reflects this distribution, showing local peaks in fluorescence at regions where VGluT1 intensity transitions between high and low. Additionally, Olig2 fluorescence dips in the central area, where VGluT1 intensity is low and MAP2 intensity peaks, as well as in the regions with high VGluT1 intensity (Figure 2B). This pattern remained until approximately P14 (Figure 2C). After the second postnatal week, oligodendrocytes were more abundant in the MSO center (Figure 2A). Correspondingly, the Olig2 profile showed an increased intensity in the somatic region, where MAP2 intensity peaks, in contrast to the dendritic regions with low MAP2 intensity (Figures 2B,C). Thus, the Olig2 profile nearly aligned with the MAP2 intensity distribution. Together, we found a nucleus-specific rearrangement of oligodendrocytes during postnatal development in the SOC.

### 3.2 Nucleus- and species-specific oligodendrocyte quantity

We observed developmental changes in oligodendrocyte cell number in SOC nuclei of gerbil (Figure 1A), related to previous findings in the MNTB (Brandebura et al., 2018; Dinh et al., 2014). To corroborate this finding, we counted the number of oligodendrocyte cell bodies within nuclear boundaries of the MNTB, MSO, LSO and dLSO, which were drawn based on VGluT1 labeling. The control region of 179 × 179 μm was assigned to a VGluT1-negative area dorsal of the MNTB. Quantification of Olig2-positive cell bodies in the selected regions between P5 and P59 showed that cell numbers in all SOC nuclei increased toward the second postnatal week reaching their maxima between P10 and P14 (Figure 3A). Due to the large variability between different sections, this increase reached significance level only in the LSO and dLSO. The subsequent decline in cell numbers continued steadily at least until P54. Comparing P5 and P54 values showed, that mature cell counts were larger only in the LSO, while in the other regions similar values were found. This data indicates that a high abundance of oligodendrocytes remained for varying time periods within the SOC nuclei.

Cell number, however, will not directly reflect cell density because SOC nuclei sizes changed during postnatal development and differed between sections. In the MNTB, MSO and LSO, nucleus size increased until P36 and remained larger compared to P5 (Figure 3B). In the dLSO, area size increased until P21, remained steady till P36 and grew again toward P54, indicating that the timing of nucleus size growth might vary between this periolivary region and other SOC nuclei. Overall, both the cell number and nuclei size showed a large variance in our samples within an age group. The control area was by definition the same size throughout development. As highlighted previously (Zacher et al., 2023), size differences between nuclei during development and their variance urged for the use of cell density to compare oligodendrocyte quantity between regions and age groups.

Cell density was calculated by dividing the count of cell number per section by the respective area size (Figure 3C). Density values showed a much smaller variance compared to that of counts. Comparing the different age groups showed that cell density was highest at P5 and remained stable for varying days in the SOC nuclei. A continuous decrease of density was observed from P10 in the MNTB, P14 in the MSO and LSO, and from P17 in the dLSO. Density values reached a steady level at P36 in the MSO, LSO and dLSO or at P54 in the MNTB that were lower compared to P5. Oligodendrocyte density in the control region was similar throughout postnatal development, except for a transient increase at P17. Comparison between nuclei revealed similar values at P5, while at P54 cell density significantly differed between binaural nuclei (MSO and LSO) and the other regions. Because cell density in the control region was rather stable during development, a nucleus-specific requirement of oligodendrocyte availability was evident that extended into periolivary regions.

To compare oligodendrocyte quantity between mammals with different brain size, we used Etruscan shrews having a considerably smaller brain size compared to gerbil. In this species, oligodendrocytes were counted in the MNTB, a highly conserved structure across species (Hilbig et al., 2009; Kulesza and Grothe, 2015; Nordeen et al., 1983; Ollo and Schwartz, 1979; Vater and Feng, 1990). Adult Etruscan shrews showed a much smaller MNTB nucleus size and a lower oligodendrocyte count compared to gerbil. However, cell density was 1.3 fold larger compared to that in the mature gerbil MNTB suggesting a species-specific oligodendrocyte density.

### 3.3 Development-dependent identification of oligodendrocytes

It was argued that oligodendrocytes can be identified more specifically by Olig2 and SOX10 co-expression (Foerster et al., 2019; Huang et al., 2023; Sock and Wegner, 2021). Since we have identified oligodendrocytes so far only by Olig2, co-labeling with SOX10 was important to validate the developmental profile of oligodendrocyte density in SOC. We, therefore, determined the overlap of the Olig2 and SOX10 labeled cell populations within a defined area of 72 × 72 μm in the MNTB, MSO, LSO and the control region (Figures 4A,B) at P5, P14, and P36. In all age groups, double-labeling and single labeling for Olig2 or SOX10 was found. The relative portion of single labeled cells was largest in the LSO at P5, where 63% of cells showed single Olig2 labeling, while <1% was solely SOX10-positive (Figure 4C). Rare single SOX10 labeled cells increased during development, while the relative portion of single Olig2 labeled cells appeared larger before compared to after hearing onset in the SOC nuclei but not the control region (Figure 4C). To verify that our previous estimates of the developmental refinement of oligodendrocytes based on Olig2 are robust, we compared the normalized densities of Olig2 labeled and Olig2/SOX10 co-labeled cells (Figure 4D). The developmental profiles of Olig2- and Olig2/SOX10-positive cells were largely similar showing a reduction in cell density to comparable levels at P36. However, at P5 in the LSO more Olig2 and at P14 in the MSO more Olig2-SOX10 co-labeled cells were detected. Thus, the described overall developmental decrease of oligodendrocyte density is valid, but cells expressing both, Olig2 and SOX10, might decrease slightly delayed compared to only Olig2 labeled cells. In contrast to gerbil, the proportion of single labeling in the mature MNTB of Etruscan shrew differed. There, a larger fraction of solely SOX10-labeled oligodendrocytes compared to Olig2-positive cells was detected (Figure 4C). The normalized density of Olig2- and Olig2/SOX10 labeled oligodendrocytes was similar and overall higher in shrews compared to mature gerbil (Figure 4D). Thus, oligodendrocyte identification with standard markers known to detect oligodendrocytes throughout development varies between age groups and species.

S100 labels oligodendrocytes from different developmental stages comprising OPCs and mature myelinating oligodendrocytes as well as myelin sheaths in the LSO, and the number of labeled cells appears developmentally regulated (Rickmann and Wolff, 1995; Dyck et al., 1993; Du et al., 2021). Yet, information on the maturation stage of S100-positive oligodendrocytes throughout the SOC and a possible developmental regulation is missing. At P5, S100 labeling was virtually absent in the SOC and only some labeled cells appeared dorsal to the MNTB (Figure 5A). Therefore, cell labeling was only quantified in P10, P14 and P54 gerbils. At P10, S100-positive cells were detected in the SOC nuclei and the control area (Figures 5A,C), and their density differed between regions being highest in the MNTB and control region. At P14 and P54, similar S100 cell densities appeared between each nucleus and the control region. Comparison of S100 cell density within regions showed that from P10 to P14, ages shortly before and after hearing onset, density only increased in the LSO and remained unchanged in the other SOC nuclei and the control region. Thereafter, S100 cell density significantly increased for all tested regions (Figure 5C). The finding that after P10, cell density was similar between SOC nuclei contrasts our data of Olig2 labeled oligodendrocytes (Figure 3C) and indicated a nucleus-independent distribution of S100-positive oligodendrocytes at mature stages. Taken together, we assume that S100 identifies cells of the same maturation stage, while Olig2 labels different subtypes of oligodendrocytes.

Given that some cells showed single Olig2 or SOX10 labeling or co-expression of both markers (Figure 4C), we aimed to investigate the distribution of S100 across different oligodendrocyte subpopulations by co-labeling of S100, Olig2 and SOX10 (Figure 4D). Moreover, to validate the assumption that S100 identifies oligodendrocytes of the same maturation stage, we labeled S100 together with MBP (Figure 4E). The majority of S100-positive cells at P10, P14 and P54 co-expressed Olig2 and SOX10, verifying their oligodendrocyte identity, and only few cells solely labeled for S100 (Figures 5B,D). At P10 and P14, S100-labeled cells constituted a minor fraction (3–27%) of all determined oligodendrocytes, while at P54 around two-third of SOC oligodendrocytes (63–74%) were S100-positive (Figure 5D). Because virtually all S100-positive axons co-labeled with MBP (97.86% at P10, 98.76% at P54, Figure 5E) and, almost all MBP-positive axons co-labeled with S100 (99.32% at P10, 99.35% at P54, Figure 5E), we propose that S100 identifies myelinating oligodendrocytes in the SOC. Thus, at P10 and P14 the majority of oligodendrocytes were non-myelinating (Olig2- and/or SOX10-positive), while in the mature age group 57–73% were of myelinating nature and oligodendrocytes of other developmental stages existed. Because in the young age group, a higher fraction of myelinating cells appeared in the MNTB compared to LSO and MSO, the timely differentiation of non-myelinating cells into mature oligodendrocytes might differ between monaural and binaural nuclei.

We observed, similar to a previous description (Jang et al., 2019), that oligodendrocytes locate in proximity of calyx-MNTB neuron synapses. To quantify the fraction of non-myelinating and myelinating oligodendrocytes at calyx synapses, Olig2 and S100 was co-labeled with VGluT1 at P10 and P54. Single Olig2 or S100-positive cells and double labeled oligodendrocytes were counted in a defined area around VGluT1-positive calyces (Figure 5F). The average number of oligodendrocytes in calyx proximity decreased from five cells at P10 to one cell at P54 (Figure 5G). In both age groups, single S100-positive cells were almost absent, while the majority of cells were solely Olig2-positive and thus non-myelinating cells (Figures 5G,H). During development, the average number of single Olig2-positive cells per calyx decreased more (4:1) compared to Olig2/S100 labeled cells (1:<1) (Figure 5G). This led to a higher proportion of double labeled, hence myelinating oligodendrocytes at P54 (Figure 5H). However, comparing synaptic with global nucleus labeling indicated that non-myelinating oligodendrocytes remained more likely at the synapse due to the relative change in ratios. At P10, the proportions of S100/Olig2 and solely Olig2-positive cells were similar at the synapse and nucleus (Figures 5D,H). By P54, one third of all Olig2-positive cells in the nucleus lacked S100, while at the synapse two thirds were S100 negative (Figures 5D,H). Thus, because a one-to-one ratio establishes at the mature calyx, the one remaining oligodendrocyte is likely a non-myelinating oligodendrocyte.

### 3.4 Developmental alterations of single oligodendrocyte processes

To determine the morphometry of single oligodendrocytes, we electroporated cells in acute brain slices within the MNTB, co-labeled these sections with NFA or VGluT1 (Figure 6A) and imaged them via confocal microscopy (Figure 6B). Cells were identified as oligodendrocytes when their processes exhibited elongated regions devoid of central labeling (Figure 6B), resembling axon ensheathment. The number of processes that gave rise to a myelin sheath decreased during development (Figure 6C, right). When all processes were counted, also somatic protrusions that did not lead to myelin sheaths, the decreasing trend was less pronounced and did not reach significance level (Figure 6C, left). The length and diameter of myelinating processes increased significantly early in development (Figure 6D). Because the main fiber bundle through the MNTB runs medio-laterally, we tested, whether the orientation of the processes showed a preference and developmental regulation (Figure 6E). Oligodendrocyte processes oriented on average medio-laterally around 45° showing no developmental change and a large variability. This variability indicated that single oligodendrocytes in the SOC myelinate axons passing in various directions (see example in Figures 6A,B). Next, because the process length increased, we analyzed whether the overall tissue area covered by a single oligodendrocyte changes during development. Therefore, the most distal points of an oligodendrocyte were connected to form a polygonal, representing its surface area, shape and orientation (Figure 6F). The area increased significantly during development showing an increase in single cell coverage (Figure 6G, left), while the orientation remained unaltered (Figure 6G, right). The roundness of the polygonal changed transiently during development (Figure 6G, middle). The 2.6 fold increase in coverage area from P10 to >P25 was larger than the increase in nucleus size (1.7 fold) or brain size (1.5 fold; 1.4 to 2.1 cm^2^) and, thus, might be adjusted to local axonal requirements and an increase of speed.

## 4 Discussion

### 4.1 Oligodendrocyte arrangement in the developing SOC

We found that oligodendrocyte localization within the developing SOC is not static. Various rearrangements take place locally and on the level of the nucleus. One such rearrangement occurred along the tonotopically arranged axonal input–output pathways. In the gerbil MNTB and LSO, after the second postnatal week, a higher oligodendrocyte density established in the lateral compared to the medial region of these nuclei, where axonal fibers exit the nuclei, consistent with previous findings in the LSO (Hafidi et al., 1994). This might be due to a structural requirement to myelinate the additional axons originating from MNTB neurons plus the passing fibers. Because oligodendrocytes myelinate fibers in both directions of their soma, a more lateral accumulation of their cell bodies is sufficient to explain the requirement for the additional myelination. In another scenario, the local, lateral increase might underlie functional requirements. Within the MNTB, and especially within its lateral region, neurons are implicated in low frequency sound processing resolving ITDs that is known to require sub-millisecond precision. Therefore, it can be hypothesized that in gerbil the local enhanced oligodendrocyte density and myelination is relevant for precision, e.g., ITD processing. This is supported by our data in Etruscan shrew and existing data in mice (Lucas et al., 2021). Both species likely lack ITD-processing, and in both species no local, lateral increase in oligodendrocytes is observed. Thus, to explain the local, lateral increase in oligodendrocytes we favor the functional over the structural explanation.

Further local, developmental oligodendrocyte rearrangements were observed in the gerbil MSO. There, somata are aligned dorsoventrally, with medio-lateral dendrites and axons that project lateral from the somatic region (Callan et al., 2021; Rautenberg et al., 2009; Kapfer et al., 2002). The glutamatergic input pattern changes shortly after hearing onset from an almost exclusive dendritic location to an additional perisomatic position (Franzen et al., 2020) depicted in the changing VGluT1 fluorescence profile. During the second postnatal week, less oligodendrocytes appeared in the central, somatic MSO region void of VGluT1 and accumulated at the VGluT1-positive distal and proximate dendritic borders and axonal origins. Thereafter, a reversed pattern established with more oligodendrocytes located near MSO somata. Thus, oligodendrocyte redistribution in the developing MSO coincides with the spatial refinement of excitatory inputs. During an overlapping period, inhibitory inputs at MSO neurons undergo a spatial rearrangement. Glycinergic synapses at the soma and dendrites present at P10 are selectively eliminated from the dendrites to establish a refined somatic location by P17 (Kapfer et al., 2002; Magnusson et al., 2005; Werthat et al., 2008). The temporal correspondence of oligodendrocytes and synaptic redistribution in the developing MSO might suggest their active involvement in synaptic remodeling. Oligodendrocytes might initially restrict excitatory synapses to MSO dendrites and sort both input types to their final destination or support the establishment of somatic synapses, e.g., by BDNF signaling (Jang et al., 2019). Moreover, because OPCs have been shown to phagocytose synapses and are, therewith, able to actively sculpt neural circuits (Buchanan et al., 2023; Buchanan et al., 2022), oligodendrocytes may eliminate inhibitory inputs from MSO dendrites. Our labelings show that in the MSO non-myelinating OPCs constitute the majority of maturation stages until P14 and a large fraction even persists thereafter, facilitating their active participation in refinement processes.

### 4.2 Developmental changes in oligodendrocyte quantity

We show that oligodendrocyte numbers in all SOC nuclei increased from P5 toward the second postnatal week and subsequently declined to a steady level around P54. An increased occurrence of oligodendrocytes from P0 to P14 was described earlier (Brandebura et al., 2018; Dinh et al., 2014) and our analysis extends and deepens these initial documentations.

Oligodendrocyte density in SOC nuclei appeared high during the first postnatal week and started to decrease at different time points thereafter, while density in the control region appeared constantly low suggesting a nucleus-specific transient requirement of oligodendrocyte availability. The observed decrease onset in oligodendrocyte density overlaps with the finalization of afferent axon refinement in the MNTB (Holcomb et al., 2013; Rodriguez-Contreras et al., 2006) and LSO (Case et al., 2011; Hirtz et al., 2012; Kim and Kandler, 2003; Walcher et al., 2011). This supports the role of oligodendrocytes in SOC circuit refinement and suggests the presence of oligodendrocyte cell types relevant for purposes other than myelination. Such cells include non-myelinating OPCs, which can phagocytose synapses to actively sculpt neural circuits (Buchanan et al., 2023; Buchanan et al., 2022). Here, we show that non-myelinating oligodendrocytes represent the main cell type until P14 and, consistent with previous findings (Lucas et al., 2021), exist throughout development.

Comparison of gerbil and Etruscan shrew, species with diverging brain size, revealed that nucleus size and oligodendrocyte count in the mature Etruscan shrew MNTB is much smaller, but their oligodendrocyte density is larger. Because body and brain size correlate (Harvey et al., 1980; Iwaniuk et al., 2005; Smaers et al., 2021), a corresponding scaling of MNTB size can be expected. Indeed, the difference in nucleus size matched that in brain size (6 mm shrew, 16 mm gerbil), both being approximately 40% smaller in shrews compared to gerbil. Differences in oligodendrocyte density between both species indicates variations in neuron and, thus, axon densities, as the number of mature oligodendrocytes is thought to match the number and length of axons (Barres and Raff, 1999; McTigue and Tripathi, 2008). Hence, the higher oligodendrocyte density in Etruscan shrew would point toward a higher neuron density in shrew compared to gerbil matching findings in the neocortex (Stolzenburg et al., 1989).

### 4.3 Oligodendrocyte identification in the developing SOC

Olig2 and SOX10 are considered specific markers for identifying oligodendrocytes throughout the postnatal period (Foerster et al., 2019; Huang et al., 2023; Sock and Wegner, 2021). At P5 in the gerbil SOC, a significant number of oligodendrocytes were detectable only with Olig2 but not SOX10. By P36, most oligodendrocytes expressed both markers, although cells positive for only Olig2 or SOX10 were present. Therefore, using SOX10 alone for oligodendrocyte quantification would have underestimated oligodendrocyte density at P5. Because only a small fraction of solely SOX10-labeled cells exists in the developing SOC, Olig2 appears as a suitable marker to quantify oligodendrocytes in the SOC. In the mature MNTB of Etruscan shrew, a higher percentage of cells were detectable with SOX10 alone, while single Olig2-positive cells were rare, indicating species-specific differences. Though the overall mechanisms of SOX10 and Olig2 in oligodendrocyte development are conserved (Fan et al., 2023; Werner et al., 2007; Ye et al., 2023), differences in genomic interactions for example might result in species-specific regulatory mechanisms of both proteins (Küspert et al., 2011).

To distinguish between oligodendrocyte maturation stages, including myelinating and non-myelinating cells, various markers have been identified (Foerster et al., 2019; Huang et al., 2023; Sock and Wegner, 2021). We showed that S100, which’s beta subunit is commonly used to label astrocytes (Brozzi et al., 2009; Hagiwara and Sueoka, 1995), identifies myelinating cells in the SOC, matching findings for S100ß in other brain regions (Dyck et al., 1993; Du et al., 2021; Deloulme et al., 2004; Santos et al., 2018). We found that prior to hearing onset, these cells were sparse and their density varied between different nuclei. At mature stages, myelinating cells became predominant, with similar densities across SOC nuclei. This however does not readily imply a homogenous myelination pattern. Because Olig2-determined oligodendrocyte densities differed between SOC nuclei at P54, we infer that S100 identifies cells of the same maturation stage, while Olig2 labels different oligodendrocyte subtypes.

The observed fraction of non-myelinating oligodendrocytes that persists in the mature SOC, might participate in refinement and plasticity processes (Jang et al., 2019; Buchanan et al., 2023; Buchanan et al., 2022; Auguste et al., 2022) and provides metabolic support to neurons (Kuhn et al., 2019). Such functions require these cells to be in close proximity to synapses (Jang et al., 2019). Using S100 as a potential marker for myelinating oligodendrocytes, our data imply that mainly non-myelinating oligodendrocytes locate in close proximity to calyx-MNTB neuron synapses before and after hearing onset. Because the developmental decrease of oligodendrocytes at calyx synapses (80%) exceeds the overall decrease of oligodendrocytes in the MNTB (60%), a targeted retraction and oligodendrocyte-to-synapse ratio can be assumed. Although astrocytes, typically expressing S100ß, also wrap axons in the MNTB, S100-positive cells at the calyx of Held can be expected to be oligodendrocytes, because axon wrapping by astrocytes drastically declines after P6 (Heller et al., 2024) and S100-positive cells become prominent only after P5.

### 4.4 Developmental alterations in oligodendrocyte process morphology

The number of myelinating oligodendrocyte processes in the MNTB decreased during development on the single cell level. During development, the number of myelinating oligodendrocyte processes in the MNTB decreased at the single-cell level. This reduction could be due to either the natural pruning of axonal branches s (Riccomagno and Kolodkin, 2015) or the maturation of cells into fully myelinating oligodendrocytes, which typically have fewer processes than premyelinating oligodendrocytes. Oligodendrocytes which do not yet sheath axons have many thin processes, which are lost as they mature (Hardy et al., 1996). Cells at P10 indeed possessed more thin processes compared to oligodendrocytes of the mature age group. This might indicate their transition from non-myelinating OPCs to mature myelinating oligodendrocytes, which is supported by our S100 labeling. The finally matured oligodendrocyte process number in the MNTB differs to other brain areas (Butt and Ransom, 1993; Osanai et al., 2017; Toth et al., 2021; Zonouzi et al., 2019) corroborating their spatial heterogeneity.

Oligodendrocyte process length is region-specific (Butt and Ransom, 1993; Toth et al., 2021; Bechler et al., 2015; Murtie et al., 2007; Ransom et al., 1991). Process lengths in our mature age group were in a similar range as recently reported internode lengths in the MNTB (Nabel et al., 2024; Ford et al., 2015). As observed in the rat optic nerve (Butt and Ransom, 1993), process length of MNTB oligodendrocytes increased during postnatal development. The increase in process length from P10 to >P25 (1.7 fold) matched the increase in nucleus size and, thus, appears associated with the ongoing maturation of auditory pathways resulting in larger distances. The MNTB-LSO projection pathway for example has not reached complete maturity at P14 (Sanes and Friauf, 2000; Sanes and Siverls, 1991). Hence, axonal pathways grow to eventually reach their target nuclei and oligodendrocyte process length increases to cover the growing distances supported by the observed developmental increase in single cell coverage.

We found that the process diameter of MNTB oligodendrocytes grows until at least P30. In line with our findings, axon and myelin thickness of afferent MNTB axons were shown to grow continuously until P25 and P35. This increase in thickness is supposed to double conduction speed of MNTB inputs following hearing onset (Sinclair et al., 2017; Nabel et al., 2024). Here, no separation between processes that myelinate afferent or efferent axons could be made. Thus, the similar developmental time course of diameter increase suggests a general oligodendrocyte process refinement throughout the MNTB.

Together, our data show that alterations in oligodendrocyte arrangement, density and morphology in SOC nuclei are closely linked to important time points during circuit establishment and refinement implicating additional functions for oligodendrocytes beyond axon myelination. The fact that morphological descriptors of oligodendrocyte processes cover a broad range of values supports the idea that oligodendrocytes represent a highly heterogeneous cell population throughout the CNS and adapt to circuit specific requirements.

## Data Availability

The raw data supporting the conclusions of this article will be made available by the authors, without undue reservation.
